# Endothelial Cell Loss, Cumulative Dissipated Energy, and Surgically Induced Astigmatism in Sutureless Scleral Tunnel Phaco-Assisted Cataract Extraction in Advanced Cataracts

**DOI:** 10.1155/2022/4272571

**Published:** 2022-05-17

**Authors:** Marwa M. Salama, Sherif A. GamalElDin, Malak I. ElShazly

**Affiliations:** ^1^Assistant Professor of Ophthalmology, Cairo University, Giza, Egypt; ^2^Professor of Ophthalmology, Cairo University, Giza, Egypt

## Abstract

**Purpose:**

To evaluate sutureless scleral tunnel phaco-assisted cataract extraction in regards to the cumulative dissipated energy (CDE) used, the resulting endothelial cell loss (ECL), and the surgically induced astigmatism (SIA) in advanced cataracts.

**Methods:**

A prospective interventional uncontrolled case series was performed. Patients with advanced cataracts according to the Lens Opacities Classification System III (LOCS III) had sutureless scleral tunnel phaco-assisted cataract extraction. They were followed up one week, one month, and 3 months postoperatively for SIA and ECL. The used CDE was recorded.

**Results:**

The study included 198 eyes: 36 eyes (18.2%) with LOCS III grade nuclear opalescence (NO4) cataracts, 102 eyes (51.5%) with LOCS III grade NO5, and 60 eyes (30.3%) with LOCS III grade NO6. Three months postoperatively, the mean SIA was 0.94 ± 0.71D. The endothelial cell density (ECD) was reduced significantly to 2341.31 ± 471 cells/mm^2^ (*p*=0.0001) with a mean ECL of 5.39%. The mean CDE and ECL% were 0.174 ± 0.46 U/S (2.07%), 0.859 ± 0.42 U/S (5.01%), and 2.306 ± 0.89 U/S (8.01%) in LOCS III grade NO4, NO5, and NO6, respectively. The overall mean CDE was 1.17 ± 0.99 U/S, which was significantly correlated with the ECL (*p*=0.0001).

**Conclusion:**

Sutureless scleral tunnel phaco-assisted cataract extraction in advanced cataracts enabled reduction in CDE with good preservation of the ECD and acceptable SIA.

## 1. Introduction

Phacoemulsification is currently the predominant technique for cataract removal worldwide with good results [1]. However, the hard nuclear cataracts require more phacoemulsification time and energy with higher complication rates [2]. Endothelial injury and cell loss in these cases remain unavoidable with a high risk of corneal decompensation in cases with borderline endothelium [3].

Femtosecond laser-assisted cataract surgery (FLACS) has recently been adopted as it proved its efficiency in reducing the endothelial cell loss and postoperative corneal edema compared to conventional phacoemulsification [4, 5]. The reduced phaco time and energy with less anterior chamber (AC) manipulations in FLACS decrease trauma to the endothelium, resulting in improved visual outcome and faster recovery [6]. However, the higher cost of FLACS still stands as an obstacle for gaining popularity as the standard technique for cataract removal, especially in the developing countries [7].

In our study, we targeted reduction of phaco time and energy in cases with advanced cataracts by doing a sutureless scleral tunnel phaco-assisted cataract surgery and studied the resulted cumulative dissipated energy (CDE), endothelial cell loss (ECL), and the surgically induced astigmatism (SIA) 3 months postoperatively.

## 2. Patients and Methods

This prospective interventional study was performed on 198 eyes of 198 patients with visually significant advanced cataracts, during the period between September 2019 and May 2020. The study was approved by the institutional review board of Dar El Oyoun Hospital, Cairo, Egypt, and was conducted in accordance with the principles of the Declaration of Helsinki. Written informed consent was obtained from all patients before surgery. Patients with a previous history of ocular surgery, trauma, or any associated ocular pathology other than cataract were excluded from the study. All patients were subjected to thorough ophthalmological examination preoperatively, including slit lamp biomicroscopy for cataract grading in accordance with the Lens Opacities Classification System III (LOCS III) [8] and anterior segment examination, applanation tonometry, indirect ophthalmoscopy, refraction using autorefractometry and uncorrected distance visual acuity (UCDVA), and best-corrected distance visual acuity (BCDVA) using the Snellen visual acuity chart. K readings and intraocular lens (IOL) power were obtained using the IOLMaster (Carl Zeiss Meditec, Jena, Germany). Endothelial cell density (ECD) was measured using a noncontact specular microscope (SP 2000P; Topcon, Tokyo, Japan) with the image-net imaging system (version 4.0; Topcon).

All surgeries were performed by a single surgeon (SG). Cases were carried out under local peribulbar anesthesia. Under complete aseptic conditions, the eye to be operated on was draped, speculum was applied, and 5% povidone iodine was instilled in the conjunctival cul-de-sac. A fornix-based conjunctival dissection was performed superiorly, and a wet field bipolar cautery was used to induce hemostasis. A scleral incision from 11 to 2 o'clock, 1.5 mm posterior to the limbus, was created and completed as a sclerocorneal tunnel up to 2 mm to the cornea using a crescent knife. Entry to the AC was performed using a 3.2 mm keratome. Viscoelastic was injected. A side port entry was made at the 3 o'clock position. Large capsulorhexis and hydrodissection were performed. Nuclear disassembly was performed by vertical chopping using a phaco probe with the combined torsional/longitudinal US mode (continuous torsional phacoemulsification with linear amplitude, 100% limits; longitudinal US linear power, 30% limits; and vacuum limit linear 450 mm Hg; Infiniti Vision System; Alcon, Fort Worth, Texas, USA) through the sclera-corneal wound, helped by using a chopper through the side port, cracking the nucleus into 4–8 fragments, according to the nucleus size and density [9]. Widening of the inner opening of the tunnel to 5 mm was achieved by using a 5 mm blunt-tip keratome. Delivery of the nuclear fragments outside the capsular bag and through the wound, assisted by viscoexpression, was performed. The remaining cortical matter was aspirated using a double-way cannula. A foldable acrylic hydrophobic IOL was implanted in the bag under viscoelastic. Viscoelastic was aspirated and the AC was irrigated, and intracameral preservative-free Vigamox (moxifloxacin 0.5%) was injected. The side port opening was sealed by stromal hydration; meanwhile, AC formation and sealing of the wound were checked. The conjunctiva was reposited to cover the wound and cauterized.

The eye speculum was removed. Tobramycin and dexamethasone eye ointment was applied, and the eye was patched. The CDE used for each patient and phaco time were documented.

Postoperative treatment included topical Zymar (gatifloxacin eyedrops 0.3%) 4 times/day continued for 6 weeks and 1% prednisolone acetate 8 times/day, tapered over the 6 weeks. Patients were followed up on the 1^st^ and 7^th^ postoperative day and later at 1 and 3 months postoperatively. One week and one month postoperatively, the BCDVA and the ECD were reported. At the last follow-up visit, UCDVA, BCDVA, ECD, and K-readings were recorded and the resulting SIA was calculated. The intraoperative and postoperative complications were also documented.

Statistical analysis was performed using IBM SPSS v21.0 statistical software (IBM Corporation, USA). Descriptive statistics were calculated, the data were summarized as mean ± SD, and frequencies were reported with numbers and percentages. Mean and standard deviations of the BCDVA were calculated after LogMAR conversion. Comparisons between the preoperative and postoperative data were carried out using the Wilcoxon signed-rank test, and comparison between more than 2 independent groups was performed by the Kruskal–Wallis test.

Nonparametric correlation between variables was performed by Spearman's correlation coefficient (*r*). The results were considered statistically significant with a *p* value ≤0.05. Astigmatism analysis was performed using the Alpins method [10]. To evaluate and describe the effect of the procedure, the surgically induced astigmatism (SIA) vector was calculated. A valid analysis was achieved by converting all refractive values (spherical equivalent (SE) and cylinder) to the corneal plane and performing all calculations based on these corneal values.

## 3. Results

One hundred ninety-eight eyes of 198 patients who met the inclusion and exclusion criteria were included in this study. Demographic data, preoperative data, and cataract grading of the patients are shown in Table 1.

Three months postoperatively, the mean LogMAR BCDVA was significantly improved to 0.16 ± 0.09 (*p*=0.001). The ECD was reduced significantly to 2341.31 ± 471 cells/mm^2^ (*p*=0.0001) with a mean ECL of 133.51 ± 77.55 cells/mm^2^ (5.39%), as shown in Table 2.

The overall mean CDE was 1.17 ± 0.99 U/S, which was significantly correlated with the ECL (*p*=0.0001). The mean phaco time was 15.67 ± 10.54 seconds.

**Figure 1:**
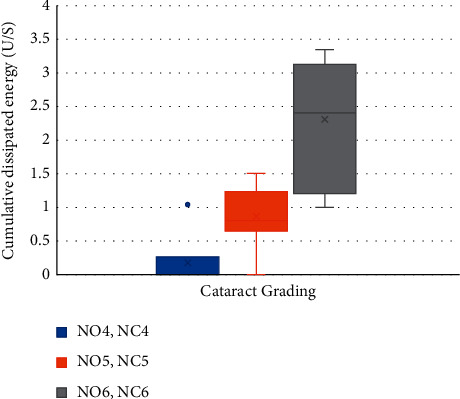
The box and whisker plot showing correlation between different cataract grades and the mean cumulative dissipated energy used (NO: nuclear opalescence; NC: nuclear color).

**Figure 2:**
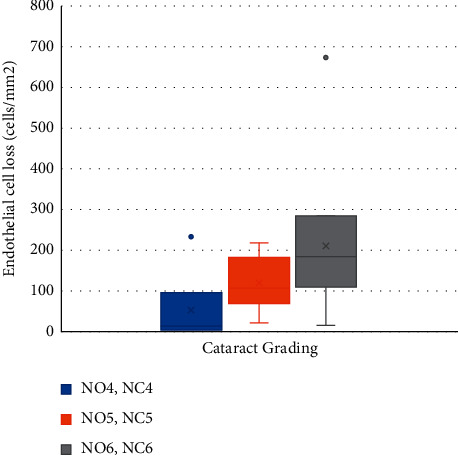
The box and whisker plot showing correlation between different cataract grades and the mean endothelial cell loss (NO: nuclear opalescence; NC: nuclear color).

Table 3 demonstrates the significant positive correlation between different cataract grades and the mean CDE (*p*=0.0001), as well as the mean ECL (*p*=0.01), as shown in Figures 1 and 2, respectively. The mean SIA and its related data are shown in Table 4.

Intraoperatively, opened posterior capsule occurred in 2 cases (1.01%), with implantation of foldable 3-pieces IOL in the sulcus. One of these 2 cases required anterior vitrectomy with scleral suturing till the IOL implantation, and then, the sutures were removed after ensuring the formation of AC with good IOP. In the other case, the opening of the posterior capsule occurred at the end of irrigation/aspiration and did not require anterior vitrectomy.

Zonular dialysis of 2 clock hours in LOCS III grade nuclear opalescence (NO) 6, nuclear color (NC) 6 case and in grade NO5, NC5 pseudoexfoliation case occurred, with no vitreous loss and did not interfere with in the bag implantation.

The aforementioned intraoperative complications had no effect on the final surgical outcome regarding BCDVA, ECL, and SIA.

No postoperative complications were encountered.

## 4. Discussion

Conventional phacoemulsification was found to reduce the endothelial cell density by 1.8% to 15% in normal eyes [1, 11, 12], especially when high US energy is required for advanced grades of cataract with higher incidence of complications [13, 14].

As a result, many research studies have been conducted to study the advantages of FLACS over conventional phacoemulsification in reducing effective phaco time (EPT) and CDE. As phaco energy and time are the most important factors affecting the corneal endothelium, femtosecond laser pretreatment for cataract surgery has been used to reduce ECL even in advanced cataract grades or Fuchs' endothelial dystrophy [2, 3, 6, 15–19].

However, these advantages are tied up by the higher cost of the FLACS technique [7]. Therefore, we aimed for an affordable technique with the reduced phaco time, energy, and ECL.

In this study, we used the sutureless scleral tunnel phaco-assisted technique in advanced cataracts and analyzed the used CDE and its effect on ECD and the resulting SIA.

This hybrid technique of phacoemulsification for breaking the large hard nucleus in the bag combined with the use of the viscoelastic dispersive material for delivery of the nucleus fragments, ensuring proper coating of the endothelial cells, added more protection to the endothelium and contributed to their preservation. It allowed the decrease of the phaco time (15.67 ± 10.54 seconds) with marked reduction of CDE (1.17 ± 0.99 U/S), even in the highest grades of cataract, as we only used the phaco energy in nucleus disassembly by quick vertical chopping, preserving the endothelial cells and minimizing their loss.

A study conducted by Yesilirmak et al. showed that their mean CDE was 5.18 ± 4.58 U/S with the Centurion active-fluidics torsional phaco machine and 7.00 ± 6.85 with the Infiniti gravity-fluidics torsional phaco machine and nuclear disassembly by the phaco chop technique in their FLACS cases [20].

In the current study, our mean CDE value was 1.17 ± 0.99 U/S and 2.3 ± 0.89 U/S in the grade NO6, NC6 cases, with an ECL of 8.01%.

Three months postoperatively, we had a mean ECL of 133.51 ± 77 cells/mm^2^ (5.39%).

In studies conducted by Krarup et al. and Conrad-Hengerer et al., their post-FLACS ECL was 11.4% with CDE of 3.78 ± 5.1 U/S using Infiniti Vision System (Alcon), and lens removal was performed by “piece by piece” technique, while Conrad-Hengerer et al. had an ECL of 8.1% using the Stellaris phacoemulsification machine (Bausch & Lomb) with the stop-and-chop technique [17, 21]. The post-FLACS ECL obtained by Yu et al. using the Stellaris system (Bausch & Lomb) with the stop-and-chop technique, one month postoperatively, was 17.4% with an average phaco time of 36.7 ± 20 sec [22].

The fact that 81.8% of our cases were of grades NO5, NC5, and NO6, NC6, can explain our resulting mean ECL, as the rest of the cases (18.2%) with grade NO4, NC4, had only a mean ECL of 2.07%. In a study conducted by Chen et al. 2017, the mean post-FLACS ECL using the Stellaris system (Bausch & Lomb), 3 months postoperatively, was 7.85%, although 95.7% of their cases were of nuclear cataract grade 4 and 4.3% were of nuclear cataract grade 5 [6].

In the present study, there was a marked reduction of the CDE and ECL during management of hard cataract grades as we applied a hybrid technique of using phacoemulsification for breaking the large hard nucleus and then removing the nuclear fragments like manual small incision cataract surgery (MSICS), thus avoiding the ECL associated with MSICS occurring due to surgical maneuvers in the AC, and making us give a thought of using this approach in cases with compromised endothelium, even with brunescent cataract.

The scleral tunnel sutureless incision in the current study resulted in the SIA of 0.94 ± 0.71 *D*, which was comparable to the postconventional phacoemulsification SIA (1.1 ± 0.9 *D*) obtained by Gogate et al. [23] but slightly higher than the SIA result of Masket et al. following 3.0 mm coaxial phacoemulsification incision 6 weeks postoperatively (0.67 ± 0.48 *D*) [24]. This could be explained by the effect of early healing of the corneal incision on the SIA compared to the scleral incision.

However, reviewing studies comparing SIA using the FLACS technique and conventional phacoemulsification showed no significant differences, with superior results than ours [25–27].

This technique requires high surgical skills with perfect experience in phacoemulsification surgery, making it unsuitable for beginners.

Creation of well-designed scleral tunnel incision and the presence of angulation to reach the anterior chamber to perform a large capsulorhexis without extension and vertical chopping especially in brunescent cataracts add further difficulties to this technique and require a relatively long learning curve. Moreover, managing vitreous loss was an added challenge due to the posteriorly located incision.

Further studies are needed with a larger number of cases, to be followed over a longer period of time, targeting cases with the compromised corneal endothelium to adequately assess the long-term effect of this technique and to confirm the data obtained.

In conclusion, sutureless scleral tunnel phaco-assisted cataract extraction used reduced phaco time and energy in eyes with high cataract grading, preserving more corneal endothelium with acceptable surgically induced astigmatism.

## Figures and Tables

**Table 1:** Demographic data, preoperative data, and cataract grading of the patients.

Parameter	Mean ± SD
Eyes (*n*)	198

Mean age (*y*)	60.57 ± 7.8

Sex (*n*)	NA
Male	90 (45.5%)
Female	108 (54.5%)

Number of eyes with cataract grade (LOCSIII)	NA
NO4, NC4	36 (18.2%)
NO5, NC5	102 (51.5%)
NO6, NC6	60 (30.3%)

Mean BCDVA (LogMAR)	1.21 ± 0.49

Mean ECD (cells/mm^2^)	2474.82 ± 486.47

Preoperative cylinder (*D*)	1.52 ± 1.05

*n* = number; *y* = years; LOCS III = Lens Opacities Classification System III; NO = nuclear opalescence; NC = nuclear color; BCDVA = best-corrected distance visual acuity; ECD = endothelial cell density; mm^2^ = millimeter square; *D* = diopter.

**Table 2:** Preoperative and postoperative mean BCDVA and ECD at 1 week, 1 month, and 3 months.

	Preoperative	1-week postoperative	1-month postoperative	3-month postoperative
*Mean BCDVA (LogMAR)*	1.21 ± 0.49	0.82 ± 0.17	0.18 ± 0.1	0.16 ± 0.09
*pvalue*	NA	(0.001)	(0.001)	(0.001)
Mean ECD (cells/mm^2^)	2474.82 ± 486	2416.08 ± 406	2386.36 ± 412	2341.31 ± 471
*pvalue*		(0.0001)	(0.0001)	(0.0001)

mm^2^ = millimeter square; BCDVA = best-corrected distant visual acuity; ECD = endothelial cell density.

**Table 3:** The correlation between different cataract grades, mean CDE, and mean ECL.

	Mean CDE (US)	Mean ECL (%)
Grade NO4, NC4	0.174 ± 0.46	52.83 ± 24.1 (2.07%)
Grade NO5, NC5	0.859 ± 0.42	120.47 ± 21.5 (5.01%)
Grade NO6, NC6	2.306 ± 0.89	204.12 ± 17.2 (8.01%)
*p* value	0.0001	0.01

CDE = cumulative dissipated energy; ECL = endothelial cell loss; NO = nuclear opalescence; NC = nuclear color.

**Table 4:** The mean preoperative and postoperative cylinder, mean SIA, SIA axis, and torque effect 3 months postoperatively (vector analysis using the Alpins method).

Parameter	Three-month postoperatively
Preoperative cylinder (*D*)	1.52 ± 1.05
Postoperative cylinder (*D*)	1.68 ± 0.98
SIA (*D*)	0.94 ± 0.71
SIA axis (°)	77.06 ± 57.12
Torque effect	0.07 ± 0.83

*D*: diopter; SIA: surgically induced astigmatism; °: degree.

## Data Availability

The data generated and analyzed during the current study are available from the corresponding author on request.

## Supplementary Materials

Supplementary Materials Supplementary video showing the sutureless scleral tunnel phaco-assisted cataract extraction in advanced cataracts.
